# Clinical Insights and Future Directions in Hypothermia for Severe Traumatic Brain Injury: A Narrative Review

**DOI:** 10.3390/jcm13144221

**Published:** 2024-07-19

**Authors:** Hitoshi Kobata

**Affiliations:** Department of Emergency and Critical Care Medicine/Neurosurgery, Osaka Medical and Pharmaceutical University, Osaka 569-8686, Japan; hitoshi.kobata@ompu.ac.jp

**Keywords:** clinical trials, evacuated hematoma, meta-analysis, therapeutic hypothermia, traumatic brain injury

## Abstract

Fever control is essential in patients with severe traumatic brain injury (TBI). The efficacy of therapeutic hypothermia (TH) in severe TBI has been investigated over the last few decades; however, in contrast to experimental studies showing benefits, no evidence of efficacy has been demonstrated in clinical practice. In this review, the mechanisms and history of hypothermia were briefly outlined, while the results of major randomized controlled trials (RCTs) and meta-analyses investigating TH for adult TBI were introduced and discussed. The retrieved meta-analyses showed conflicting results, with a limited number of studies indicating the benefits of TH. Some studies have shown the benefits of long-term TH compared with short-term TH. Although TH is effective at lowering elevated intracranial pressure (ICP), reduced ICP does not lead to favorable outcomes. Low-quality RCTs overestimated the benefits of TH, while high-quality RCTs showed no difference or worse outcomes with TH. RCTs assessing standardized TH quality demonstrated the benefits of TH. As TBI has heterogeneous and complicated pathologies, applying a uniform treatment may not be ideal. A meta-analysis of young patients who underwent early cooling and hematoma removal showed better TH results. TH should not be abandoned, and its optimal usage should be advocated on an individual basis.

## 1. Introduction

Traumatic brain injury (TBI) is a leading cause of injury-related deaths and disabilities worldwide, exerting a devastating impact on patients and their families [1]. One recent systematic review reported that an estimated sixty-nine million individuals worldwide suffer from TBI from any cause each year. Although the vast majority of these are mild (81%) or moderate (11%) [2], severe TBI nevertheless constitutes a significant health and socioeconomic problem.

There is currently no available treatment for primary brain injury, defined as the direct destruction of the brain parenchyma and blood vessels. Therefore, treatments for TBI focus on mitigating secondary injury, which is triggered by a cascade of destructive events and processes beginning at the cellular level within minutes to hours following the initial injury. The duration and magnitude of the secondary injury cascade are highly variable depending on the TBI subtype. Excitotoxicity, neuroinflammation, apoptosis, free radical production, seizure activity, blood/brain barrier disruption, blood vessel leakage, and cerebral thermopooling may all develop to varying degrees [3]. Accordingly, TBI is termed “the most complicated disease of the most complex organ of the body” [4].

To date, no specific neuroprotective pharmacological treatment options with proven clinical efficacy are available for patients with TBI [1]. Importantly, all the harmful processes are temperature-dependent, meaning that they are all stimulated by fever and can be mitigated or blocked by hypothermia treatment [3]. Early preclinical studies have shown that a slight reduction in brain temperature after moderate-to-severe TBI reduces histopathological damage and neurological deficits. Investigative clinical studies have also reported reductions in multiple post-traumatic attenuated secondary injury mechanisms [5]. However, numbers of clinical studies have failed to demonstrate the benefits of hypothermia.

## 2. Methods

This study comprises three sections: a narrative review on neuroprotection and history of therapeutic hypothermia (TH), a summary of prior meta-analyses, and a meta-analysis of preoperative early TH for young patients with surgically evacuated hematoma.

A systemic search for Pubmed, Medline, and the Cochrane Central Register of Clinical Trials was performed from 1 January 2000 to 12 December 2023 using the following search terms: “hypothermia OR cooling” AND “traumatic brain injury OR TBI” AND “RCT”. The author also hand-searched the bibliographies of relevant citations and reviews. The analyses were referenced to the guidelines of the Preferred Reporting Items for Systematic Reviews and Meta-Analysis Protocols (PRISMA); however, this study was conducted by a single author and does not fulfill the PRISMA requirement, and was not registered in the International Prospective Register of Systematic Reviews (PROSPERO). 

The author conducted a meta-analysis of the retrieved literature on young patients with acute subdural hematoma who underwent the preoperative induction of hypothermia and early surgery using Review Manager (RevMan, Cochrane Collaboration, London, UK, version 5.3). 

## 3. Terminology

TH has been widely used for patients with various types of severe brain injury; however, the definition of “mild”, “moderate”, and “deep” hypothermia shows some discrepancies between studies. To avoid confusion related to these terms, five intensive care societies sponsored an expert review and analyzed the existing knowledge. The jury opined that the term “targeted temperature management (TTM)” should replace “therapeutic hypothermia”, and that the descriptions should be replaced with explicit TTM profiles [6]. In this study, TH was used as the TTM at 32–34 °C unless otherwise stated. 

## 4. Mechanism of Hypothermic Neuroprotection

**Figure 1:**
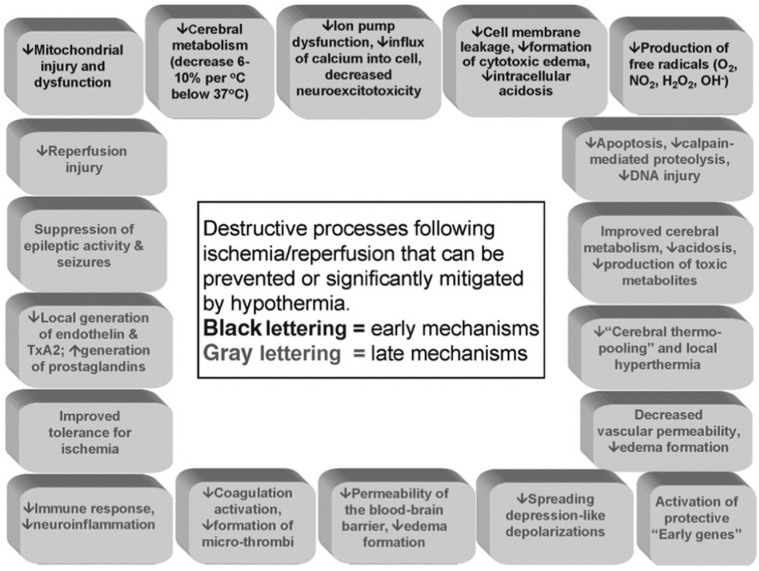
Schematic depiction of the mechanisms underlying the protective effects of mild-to-moderate hypothermia. TxA2, thromboxane A2. Reprinted from Polderman KH [3], with permission from Wolters Kluwer.

Cerebral metabolism decreases by 6% to 10% for each 1 °C reduction in body temperature. Thus, early studies used profound hypothermia below 30 °C as lower temperatures were believed to be more effective. Currently, it is recognized that reducing body temperature by a few degrees can protect the brain through various early and late mechanisms of action (Figure 1) [1].

The broad range of beneficial effects of hypothermia includes the inhibition of various destructive processes following ischemia/reperfusion injury, including ion pump dysfunction and neuroexcitotoxicity, free radical production, mitochondrial injury, cell membrane leakage, formation of cytotoxic edema, and intracerebral acidosis. The late mechanisms of TH include the inhibition of apoptosis, calpain-mediated proteolysis, reduction in vascular permeability, blood/brain barrier disruption, and edema formation.

## 5. History of Therapeutic Hypothermia in Brief

The documentation of the therapeutic effects of hypothermia can be traced back several millennia. The earliest recorded evidence of the use of cooling for disease was found in the Edwin Smith Papyrus, written in ancient Egypt. More than a millennium later, Hippocrates described the use of whole-body cooling in patients suffering from tetanus [7,8], and local cooling with ice and snow before operation work [9,10]. Areteus, a Greek physician in the second century AD, recommended prompt action and burr hole opening to remove hematomas, diuretics, and hypothermia for brain injury [11]. Thereafter, the beneficial effects of cooling have been repeatedly reported from the Renaissance until the 20th century [7].

In 1938, following vigorous laboratory investigations, Temple Fay first induced generalized refrigeration in a young woman with metastatic breast cancer who experienced systemic pain due to widespread metastasis [12]. Subsequently, the therapeutic use of hypothermia in patients with TBI was first reported in 1943 in a 22-year-old woman suffering from a cerebral contusion and laceration who had remained unconscious and developed a fever above 40 °C with tachycardia and tachypnea. She remained in a coma with a high fever around 40 °C despite local cooling. Finally, 4 weeks after the injury, she received whole body refrigeration at 33 °C for 48 h. She recovered fully and returned to work seven months later [13]. Fay et al. subsequently applied generalized refrigeration to 124 patients and developed a local cooling device [14]. 

In the 1950s, following experimental evidence showing the beneficial effects of hypothermia on brain protection [15], it was used in cardiac surgery [16], cerebral aneurysm surgery [17], and resuscitation [18]. Although early experiments investigating hypothermia appeared promising, deep hypothermia ≤30 °C aiming at lowering metabolism was abandoned because of serious adverse events [7,8,10].

## 6. Reviving Clinical Use of Hypothermia for Severe Traumatic Brain Injury

A revival of hypothermia therapy began in 1987 following the finding that lowering the brain temperature by only a few degrees conferred a marked brain-protective effect in rat ischemic models [19]. Many preclinical studies have since reported the efficacy of mild hypothermia in various aspects. These studies have encouraged the clinical use of TH in the treatment of various severe brain injuries. 

In the 1990s and the early 2000s, TH was widely used to treat severe TBI. In 1993, the first randomized controlled trials (RCTs) on TH for TBI were published [20,21,22]. Marion et al. used TH at 32 °C to 33 °C for severe TBI patients. Forty consecutive patients aged 16–75 years with GCS scores of 3–7, admitted between February 1991 and August 1992, were randomized to either the TH or normothermia group. TH was initiated within a mean of 10 h after injury and maintained for 24 h, after which the patients were rewarmed to 37 to 38 °C over 12 h. TH significantly reduced intracranial pressure (ICP) and cerebral blood flow (CBF) during cooling. This study showed a trend toward better outcomes in the TH group than in the normothermia group, without increased systemic complications [20]. Clifton et al. further conducted a phase II study on TH enrolling 46 patients with severe TBI. Patients aged 16 to 60 years with GCS scores of 4–7 were randomized to TH (32 to 33 °C) or standard management (37 °C). Cooling was begun within 6 h of injury using cooling blankets and the patients were rewarmed at a rate of 1 °C/4 h after maintaining 33 °C for 48 h. TH was associated with improved neurologic outcomes with minimal toxicity [21]. Shiozaki et al. reported that TH at 34 °C for 48 h significantly improved survival rate and reduced ICP, CBF, and cerebral metabolic rates for oxygen (CMRO_2_) for TBI patients with a GCS score of ≤8 and ICP > 20 mmHg [22]. They later recommended normothermia, in which the ICP could be maintained at <20 mmHg using conventional therapies [23].

A clinical study on severe TBI showed that TH reduced CMRO_2_ by approximately 45% without inducing significant changes in cerebral blood flow (CBF) or normalized cerebral metabolic rate of lactate, thereby preventing secondary brain damage [24]. TH reduces prostanoid production after TBI, thereby attenuating the imbalance between thromboxane A_2_ and prostaglandin I_2_ and improving outcomes [25].

## 7. Major Phase III Randomized Controlled Trials

**Figure 2:**
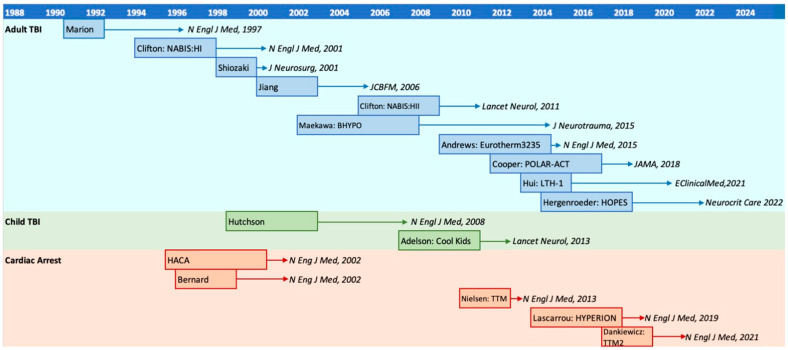
Major randomized controlled trials of therapeutic hypothermia for severe traumatic brain injury and post-cardiac arrest patients [26,27,28,29,30,31,32,33,34,35,36,37,38,39,40,41,42]. Boxes indicate recruitment periods; arrows point to the year of publication.

Based on early studies that demonstrated the potential benefits of TH in TBI, numerous phase III trials on the topic have been planned since the 1990s. Figure 2 provides an overview of the major RCTs on TH for adult TBI [26,27,28,29,30,31,32,33,34,35], together with child TBI [36,37,38], and postcardiac arrest [39,40,41,42,43]. Patient recruitment periods and years of the publication are shown.

The characteristics of the RCTs on adult TBI are summarized in Table 1. Contrary to expectations, most studies have failed to demonstrate the efficacy of TH. A summary of each RCT is provided below.

### 7.1. Marion1997

In one single-center RCT conducted in Pittsburgh, USA, TH (a temperature of 32 or 33 °C) for 24 h, initiated a mean of 10 h after severe TBI, significantly improved the outcomes at 3 and 6 months in patients with GCS scores of 5 to 7, but not in those with GCS scores of 3 to 4. TH did not increase the incidence of complications. Therefore, TH appears to be a promising treatment option for severe TBI [26].

### 7.2. Clifton: NABIS:H

This landmark multicenter RCT, involving 11 sites across the USA, assigned 193 patients to the control group and 199 to the TH group (33 °C). The mean time from injury to achieving the target temperature of 33 °C was 8.4 ± 3.0 h in the TH group. 

The study revealed no differences between the TH and normothermia groups in the primary outcome measure; 57% of the patients in both groups had a poor outcome (severe disability, vegetative state, or death), while mortality was 28% in the TH group and 27% in the normothermia groups. The lack of the benefits of TH in severe TBI had a profound impact on the management strategies. However, a significant decrease in poor outcomes was noted among the patients ≤45 years who had hypothermia on admission; 52% of those assigned to the TH group had poor outcomes, as compared with 76% of the normothermia group (*p* = 0.02) [27]. Poor outcomes ranged widely in low-enrollment centers, and the participation of small centers resulted in an increase in the intercenter variance and diminished the quality of the data [44]. This study was criticized for its slow achievement of the target body temperature and unstable circulatory dynamics, which led to future studies.

### 7.3. Shiozaki

In this study conducted at 11 medical centers in Osaka, Japan, 91 TBI patients with GCS scores ≤8 were assigned to either the TH group (45 patients) or the normothermia group (46 patients), provided that ICP remained <25 mmHg after the conventional ICP reduction therapies. Using a cooling mat, the TH group was maintained at 34 °C for 2 days and then rewarmed at a rate of 1 °C/day, whereas the normothermic group was maintained at 37 °C for 5 days. 

Although there was no difference in the outcomes between the two groups, complications were significantly more frequent in the TH group. This study concluded that TH should not be used to treat patients with severe TBI in whom ICP can be maintained at <25 mmHg using conventional therapies [28].

### 7.4. Jiang

At three medical centers in China, 215 patients aged 18–45 years with an admission GCS score ≤8 within 4 h after injury, frontotemporoparietal contusion with midline shift >1 cm, and ICP >20 mmHg were randomly divided into a long-term TH group (*n* = 108) and short-term mild TH group (*n* = 107). When the patient’s rectal temperature reached 33 °C to 35 °C, this temperature was maintained for 5 ± 1.3 days for the long-term TH group and 2 ± 0.6 days for the short-term TH group.

Favorable outcomes at 6 months were observed in 43.5% of the long-term TH group and 29.0% of the short-term TH group (*p* < 0.05). ICP significantly rebounded after rewarming in the short-term TH group but not in the long-term TH group (*p* < 0.05). No significant difference in the frequency of complications was noted between the groups [29].

### 7.5. Clifton: NABIS:H II

This study aimed to assess whether the very early induction of hypothermia in patients with severe TBI improved outcomes, involving more rapid induction and the strict maintenance of TH levels under stable hemodynamics. Ninety-seven patients (fifty-two in the TH group and forty-five in the normothermia group) aged 16–45 years were enrolled from six sites in the USA. The mean time to reach 35 °C in the TH group was 2.6 h, and to 33 °C was 4.4 h. Body temperature was precisely controlled by automated temperature feedback and adjustment systems using gel pads. 

The study, again, showed no differences in poor outcomes (60% vs. 56%, *p* = 0.67) or mortality (23% vs. 18%, *p* = 0.52) between the TH and normothermia groups. However, the patients who underwent the surgical removal of intracranial hematomas with hypothermia had significantly fewer poor outcomes than the patients who had normothermia (*p* = 0.02). Furthermore, there was weak evidence that the patients with diffuse brain injury treated with TH had poorer outcomes than the patients in the normothermic group (*p* = 0.09). However, this trial did not confirm the utility of TH as a primary neuroprotective strategy in patients with severe TBI [30].

### 7.6. Maekawa: B-HYPO Study

The B-HYPO study, which involved 36 hospitals in Japan, was conducted between December 2002 and September 2008. This study aimed to avoid the limitations of the previous studies by implementing the following rules: patients were cooled as soon as possible, hypothermia was maintained for at least 72 h while ICP was in the normal range, and patients were rewarmed at a rate of <1 °C/day with strict hemodynamic monitoring. An arterial catheter, a pulmonary arterial catheter, and an ICP-monitoring probe were inserted to maintain optimal hemodynamic status and ICP. Thus, this study only recruited patients with TBI and GCS scores of 4–8, aged 15–69 years, who were able to undergo cooling within 2 h after injury. The patients were allocated to either the TH (32–34 °C) or fever control (35.5–37.0 °C) groups at a ratio of 2:1. Core body temperature was primarily measured using a thermistor coupled to an internal jugular venous catheter, and the jugular venous oxygen saturation (SjO_2_) was continuously monitored. Biochemical data were recorded before and after the induction of hypothermia and rewarming. The CT images of all the patients were collected and classified according to the Traumatic Coma Data Bank (TCDB) classification. The principal investigator conducted site visits to each participating hospital for quality control. 

The target sample size was set at 300 patients, but enrollment slowed after the amendment of the Japanese Road Traffic Law in 2007 to make drinking and driving strictly punishable. Eventually, 98 patients were enrolled in the TH group and 50 in the temperature control group. The overall rates of poor neurological outcome were 53% and 48% in the TH and fever control groups, respectively. This study concluded that tight hemodynamic management and slow rewarming, together with prolonged TH for severe TBI, did not improve neurological outcomes or the risk of mortality compared to strict temperature control [31].

A dozen post hoc analyses were published from the data obtained in the B-HYPO study, the results of which are as follows: Diverse effects of TH were observed based on TCDB classification. Increased favorable outcomes in young patients (≤50 years old) with evacuated mass lesions in the TH group (77.8%) compared with the fever control group (33.3%), whereas the patients with diffuse injury III who were treated with TH had significantly higher mortality than the patients treated with fever control [45]. Among the patients with an Abbreviated Injury Scale score of 3–4, the fever control group demonstrated significantly lower mortality and a trend toward more favorable outcomes compared to those of the TH group [46]. Based on the analysis of the initial potassium level, fever control may be considered instead of TH in patients with normokalemia upon admission [47]. Initial stress hyperglycemia was sustained in the TH group compared to the fever control group. Blood glucose levels on the day after admission were significant prognostic indicators in both the TH and control groups [48]. Early-stage hyperoxia was associated with favorable neurological outcomes and survival [49]. TH did not negatively affect the outcomes in patients with coagulopathy and severe TBI [50]. Slow rewarming for >48 h may improve the neurological outcomes of prolonged TH in patients with TBI and evacuated hematomas [51]. A mild decrease in heart rate during the early phase of TH following tachycardia at admission could predict unfavorable neurological outcomes [52]. A reduction in the difference between the mixed venous oxygen saturation (SvO_2_) and jugular venous oxygen saturation (SjvO_2_) on day three was associated with high mortality [53]. High hemoglobin levels during the early phase were also significantly associated with favorable neurological outcomes [54]. Among the young adults (≤50 years) who underwent early surgical evacuation for acute subdural hematoma (ASDH), the TH group had better outcomes than the normothermia group despite similar CT findings [55]. The temperature difference between the jugular bulb and pulmonary artery (ΔTjb-pa) trended significantly higher in the favorable outcome patients than in the unfavorable outcome patients throughout the 120 h following the onset of severe TBI. The variation in Tjb-pa from 0 to 72 h was significantly lower in patients with favorable outcomes [56].

### 7.7. Andrews: Eurotherm3235

The Eurotherm 3235 Trial was an international, multicenter RCT that examined the effects of titrated TH (32 to 35 °C) for intracranial hypertension. Adult TBI patients with ICP >20 mmHg despite mechanical ventilation and sedation were assigned to either the control or TH group. The study enrolled 387 patients, of whom 54% were in the control group and 44% were in the TH group. More than 90% of the patients in both groups were enrolled >12 h after the injury; however, there were no significant between-group differences according to the time from injury to the initiation of hypothermia (<12 or ≥12 h).

Favorable outcomes (Extended Glasgow Coma Scale [GOS-E] score of 5–8, indicating moderate disability or good recovery) occurred in 25.7% of the patients in the TH group and 36.5% of the control group (*p* = 0.03). The risk of death (hazard ratio, 1.45; 95% CI, 1.01 to 2.10; *p* = 0.047) was superior in the control group; further, serious adverse events were reported more frequently in the TH group than in the control group (33 vs. 10 events). TH plus standard care successfully reduced ICP but did not improve functional recovery compared with standard care alone [32].

TH is an effective addition to ICP management that can reduce the number of hyperosmolar therapies required [57]. However, later proportional hazard analysis for death indicated that TH, as a first-line measure to reduce ICP to <20 mmHg, is harmful in patients with a lower severity of injury, while no clear benefit exists in patients with more severe injuries [58]. 

### 7.8. Cooper: POLAR-ACT

The Prophylactic Hypothermia Trial to Lessen trAumatic bRain injury—randomized controlled trial (POLAR-RCT), conducted in six countries, recruited 511 patients: 266 in the TH group and 245 in the normothermia group. Prophylactic TH targeted the early induction of hypothermia (33–35 °C) for at least 72 h, and up to 7 days if ICPs were elevated. Eligible patients aged 18 to 60 with a GCS score ≤8 were recruited. 

Although TH was initiated rapidly after injury (median time, 1.8 h), the time to reach the final temperature target of 33 °C took a median of 10.1 h (IQR, 6.8 to 15.9). A total of 85 evaluable patients (33%) in the TH group received hypothermia for less than 48 h (33 °C–35 °C), and 27% of the patients in the TH group never reached the final target temperature of 33 °C. Favorable outcomes (GOS-E score, 5–8) at 6 months occurred in 48.8% of the TH group and 49.1% of the normothermia group (risk difference, 0.4% [95% CI, −9.4% to 8.7%]; relative risk with TH, 0.99 [95%CI, 0.82–1.19]; *p* = 0.94). 

Compared to those with normothermia, early prophylactic TH did not improve neurological outcomes at 6 months. No significant interactions were noted between the treatment group and any of the pre-specified subgroups: the presence of surgically evacuated cranial hematomas and any intracranial hematoma (surgically evacuated or not). The patients’ age and timing of surgery in relation to body temperature were not mentioned [33].

### 7.9. Hui: LTH-1

The Long-Term Hypothermia trial (LTH-1), conducted in 14 hospitals in China, was a prospective multicenter RCT conducted to examine the safety and efficacy of hypothermia in adults with severe TBI. Eligible patients included those aged 18–65, with a GCS score of 4 to 8, and an initial ICP ≥25 mmHg. Patients were randomly assigned to the long-term TH group (34–35 °C for 5 days) or normothermia group at 37 °C. 

There were no differences between the groups in terms of favorable outcomes or mortality. However, TH significantly increased favorable outcomes over the normothermia group in patients with an initial ICP ≥30 mm Hg (60.8% and 42.7%, respectively; OR 1.861, 95%CI 1.031–3.361; *p* = 0.039). Of note, ICH removal was conducted in 91.8% of the normothermia group and 92.3% of the TH group, and decompressive craniectomy was performed in this order in 71.2% and 68.0% of the patients, respectively [34].

### 7.10. Hergenroeder: HOPES

The HypOthermia for Patients requiring Evacuation of Subdural hematoma (HOPES) trial was a multicenter RCT designed based on previous studies, in which the early induction of TH and early hematoma removal in young adults provided favorable outcomes. This RCT was conducted in the USA and Japan, enrolling patients with ASDH requiring evacuation within 6 h of injury. Patients in the TH group were cooled by an endovascular device to reach 35 °C by the time of dural opening and sustained for 48 h. Patients in the control group were maintained at 37 °C. 

The trial design aimed to enroll 120 patients; however, due to slow accrual, an early futility interim analysis was added after 31 participants completed the 6-month follow-up. There were no significant differences in favorable 6-month GOS-E between the TH and the normothermia groups (6 of 16, 38% vs. 4 of 16, 25%; odds ratio 1.8 [95% confidence interval 0.39 to ∞], *p* = 0.35) in this analysis. The plasma levels of glial fibrillary acidic protein and ubiquitin C-terminal hydrolase did not differ between the two groups [35].

## 8. Review of Prior Meta-Analyses

A PubMed/MEDLINE literature search and citations from references yielded 25 meta-analyses assessing TH for adult TBI [59,60,61,62,63,64,65,66,67,68,69,70,71,72,73,74,75,76,77,78,79,80,81,82,83]. The highlights of each study are summarized in Table 2. These meta-analyses have found conflicting results, with few studies indicating the benefits of TH. However, some studies have shown the benefits of long-term TH compared to short-term TH [60,62,64]. TH is effective in lowering elevated ICP [61,65]; however, decreased ICP does not result in favorable outcomes [32].

Several studies have previously reported on the quality of RCTs, with results showing that low-quality RCTs overestimate the benefits of TH, while high-quality RCTs showed no difference in outcome between the TH and the normothermia groups, or even worse outcomes in the TH group [63,68,69,78,79,81,83]. Indeed, two RCTs with a low risk of bias [31,32] showed significantly higher mortality, poorer outcomes, and an equal incidence of new-onset pneumonia in the TH group. In contrast, other RCTs with a high risk of bias showed the opposite, with higher mortality and worse outcomes, but fewer new pneumonia cases in the control group [77].

Several studies have addressed the heterogeneity of TBI [43,77]. One study used a cooling index calculated from the target cooling temperature, cooling duration, and speed of rewarming to standardize and assess the quality of TH. Although inter-study heterogeneity was high, TH was beneficial in severe TBI only if the cooling index was sufficiently high. As independent factors, milder and longer cooling, and rewarming at <0.25 °C/h were associated with better outcomes [78]. A cooling index-based meta-analysis, including the recent POLAR-ACT study, strengthened the results regarding the benefits of TH [83].

## 9. A Meta-Analysis of Young Patients with Surgically Evacuated Hematoma

Animal experiments investigating ASDH have shown brain swelling after hematoma removal [84], while early preoperative TH reduced ischemia/reperfusion injury following surgical evacuation [85]. Early TH may therefore offer potential benefits in attenuating ischemia/reperfusion injury in patients requiring ASDH removal [86]. In the existing RCTs, the benefits of early TH have also been suggested for young patients with evacuated mass lesions, although individual RCTs included only a small number of eligible patients [44,45,55]. Thus, a meta-analysis including the NABIS:H I, NABIS:H II, B-HYPO, and HOPES trials was performed.

**Figure 3:**
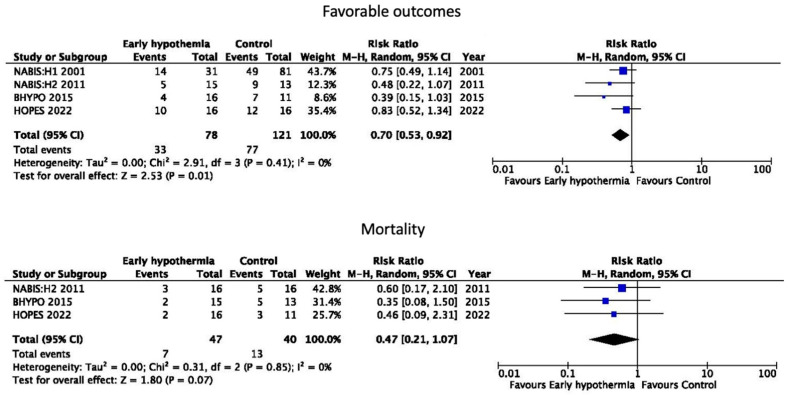
Meta-analysis of randomized controlled trials on early surgery and early hypothermia for acute subdural hematoma in young adults [27,30,31,35].

The resulting forest plot showed a significant increase in favorable outcomes in the TH group compared with the control group (RR = 0.70, [95% CI = 0.53, 0.92], *p* = 0.01). Although the mortality rates for this subgroup were not described in NABIS:H I, a trend toward lower mortality rates was observed in the TH group. (PR = 0.47, [95% CI = 0.21, 1.04], *p* = 0.07) (Figure 3). The assessment of the risk of bias is shown in Table S1. Although some differences in age and time to surgery were noted in each RCT (age ≤ 45, the induction of HT to 35 °C within 1.5 h of surgery start time in NABIS:H I and II study [44]; age ≤ 50, time to 35.5 °C 280 min in B-HYPO study [55]; average age of 43.9, 35 °C prior to dura opening in HOPES trial [35]), cooling was commonly initiated early before surgery. The meta-analysis indicated that TH would be suitable for the following patients: (1) relatively young, (2) with evacuated mass lesions, (3) with early cooling, and (4) with early hematoma evacuation.

## 10. Limitations of RCTs

Similar limitations were noted across most existing large multicenter RCTs, including a variable timing of TH initiation, poor adherence to the temperature range, different rewarming rates, a variable duration of hypothermia, and poor design and methodology [87]. Furthermore, a few essential issues have not been discussed in the existing RCTs. The first concern is the localization of brain injury. As the brain is functionally localized, functional recovery is closely dependent on the damaged region. Favorable outcomes seem unlikely in patients with severe damage to the eloquent areas. Although it would be challenging to conduct RCTs on this topic, the topographical assessment of brain damage is an essential concern. The second concern is rehabilitation. Early and appropriate rehabilitation positively affects patient recovery. The extent and duration of rehabilitation during and after TH are therefore likely to affect patient outcomes. The third is management strategies other than TH, although it may be impractical to standardize all the management procedures and medications across facilities.

## 11. Future Prospects

A systematic review and meta-analysis of experimental TBI showed that TH appeared to be an effective treatment, although it should be noted that these studies had limitations in terms of quality and design [88]. Discrepancies between experimental and clinical approaches should therefore be considered when translating animal experiments into clinical practice. For example, TH was introduced very early in animal studies compared to in clinical trials. This is important as the maxim “time lost is brain lost” commonly used to describe stroke also applies to TH for severe TBI. 

The essential question in the field of TH is “how early, how deep, how long, and how to rewarm”, which unfortunately remains unresolved. The specific pitfalls associated with the clinical management of TH, such as stress-induced insulin-resistant hyperglycemia and unstable systemic circulation, require particular attention [89]. One umbrella review of the treatment options for TBI indicated that TH is the only clinical practice with evidence of benefit [90]. Therefore, TH should not be abandoned in the treatment of severe TBI with hematomas. However, a one-size-fits-all treatment is not applicable to patients with severe TBI composed of heterogeneous lesions. Biomarkers may be valuable for stratifying the severity of TBI and assessing the effects of TH. Patients with severe TBI require individualized treatment for the underlying pathophysiology of brain injury. The meticulous management of neurocritical care is also essential for the successful completion of TH with minimal adverse events.

## Figures and Tables

**Table 1:** Characteristics of major randomized controlled trials for severe adult traumatic brain injury.

First Author	Year	Recruitment	Site	Number of Pts		Inclusion	Age	Induction	Temp	Duration	Rewarming	Methods	Outcome
(Study)		Period		TH	Control	Criteria	(Years)		(°C)	(h)			
Marion [26]	1997	1991.2 –1994.9	1	40	42	GCS 3–7	16–75	Immediately	33	24	<1 °C/h	Cooling blanket	TH better for pts with GCS 5–7
Clifton [27] (NABISH:H I)	2001	1994.10 –1998.5	11	199	193	GCS 3–8	16–65	<8 h	33	48	0.5 °C/2 h	Cooling pad	NS
Shiozaki [28]	2001	1998.2 –2000.1	11	45	46	GCS 3–8 and ICP <25 mmHg		As quickly as possible	34	48	1 °C/d	Cooling blanket	TH better
Jiang [29]	2006	2000.5 –2003.5	3	108	107	GCS 3–8	18–45	<4 h	33	48 or 120	<1 °C/h	Cooling blanket	TH better
Clifton [30] (NABISH:H II)	2011	2005.12 –2009.6	6	52	45	GCS 3–8	19–45	<2.5 h	33	48	0.5 °C/2 h	Gel pad with thermal feedback	NS
Maekawa [31] (BHYPO)	2015	2002.12 –2008.9	36	98	50	GCS 4–8	15–69	<6 h	32–34	≥72	1 °C/d	Cooling blanket	NS
Andrews [32] (Eurotherm3235)	2015	2009.11 –2014.10	47	195	192	ICP >20 mmHg	Legal age for consent—<65	<10 d	32–35	>48	0.25 °C/h	The usual cooling technique of each site	Control better
Cooper [33] (POLAR-ACT)	2018	2010.12 –2017.11	7	266	245	GCS 3–8	18–60	Prehospital and ED	33	≥72	0.25 °C/h	Cooling blanket	NS
Hui [34] (LTH-1)	2021	2013.6 –2015.12	14	156	146	GCS 4–8 and ICP ≥25	18–65	<24 h	34–35	120	<0.5 °C/4 h	Cooling blanket	TH better
Hergenroeder [35] (HOPES)	2022	2014.5 –2018.6	15	16	16	GCS Motor score ≤5 and ASDH	22–65	<6 h, ≤35 °C before dural incision	33	48–120	0.25 °C/h	Intravascular catheter with thermal feedback	NS

Abbreviations. ASDH, acute subdural hematoma; ED, emergency department; GCS, Glasgow Coma Scale; ICP, intracranial pressure; NS, not significant; pts, patients; TH, therapeutic hypothermia.

**Table 2:** Results of the meta-analyses of therapeutic hypothermia for severe traumatic brain injury in adults.

Author	Year	Included Trials	Number of Patients	Conclusions
Harris [59]	2002	7	668	TH showed no benefit in GOS, ICP, pneumonia, cardiac arrhythmia, or prothrombin time, but was associated with elevated partial thromboplastin time.
McIntyre [60]	2003	12	1069	TH to a target temperature between 32 °C and 33 °C, a duration of 24 h, and rewarming within 24 h were all associated with reduced risks of poor neurologic outcome compared with normothermia. TH longer than 48 h was associated with reduced risks of death and of poor neurologic outcome.
Henderson [61]	2003	8	748	No clear evidence of lower mortality rates was found in unselected TBI patients. Prolonged TH may confer a benefit, particularly in patients with elevated ICP refractory to conventional manipulations.
Peterson [62]	2008	13	1339	TH may reduce the risk of mortality and improve the prospects of a favorable neurological outcome, particularly when maintained for ≥48 h, and when used in patients who respond well to the standard measures of ICP control besides high-dose barbiturates.
Sydenham [63]	2009	23	1614	There is no evidence that TH is beneficial in the treatment of TBI. TH may be effective at reducing death and unfavorable outcomes, but a significant benefit was only found in low-quality trials.
Fox [64]	2010	12	1327	Short-term TH showed no improvement in mortality or neurological outcomes. Long-term or goal-directed TH reduced mortality and increased good neurological outcomes. Early prophylactic mild-to-moderate TH decreased mortality and improved the rates of good neurologic recovery.
Sadaka [65]	2012	18	1773	TH was effective in controlling ICP in all the studies. In the 13 RCTs, ICP in the TH group was always significantly lower than ICP in the normothermia group. In the five observational studies, ICP during TH was always significantly lower than prior to inducing TH.
Georgiou [66]	2013	18	1851	TH was associated with cerebrovascular disturbances on rewarming and possibly with pneumonia. No benefit on mortality or neurological morbidity could be identified in TH.
Li [67]	2014	13	1152	TH may be effective at reducing death and unfavorable clinical neurological outcomes, but this difference is not statistically significant, except for decreasing the mortality in Asian patients.
Sandestig [68]	2014	19		Two out of fourteen studies on adult TBI reported a tendency of higher mortality and worse neurological outcomes, four reported lower mortality, and nine reported favorable neurological outcomes with TH. The best-performed RCTs showed no improvement in outcome by TH.
Crossley [69]	2014	20	1885	TH may achieve benefits. The majority of the trials were of low quality, with an unclear allocation concealment. Low-quality trials may overestimate the effectiveness of TH versus standard care.
Madden * [70]	2015	16	ND	Fever avoidance resulted in positive outcomes, including the decreased length of stay in the ICU, mortality, the incidence of hypertension, elevated ICP, and tachycardia. Hypothermia on admission correlated with poor outcomes. Controlled normothermia improved surrogate outcomes. Prophylactic TH is not supported.
Dunkley [71]	2016	8	689	TH is reported to be effective at lowering ICP; however, its efficacy in improving neurological outcomes is not fully demonstrated. TH had increased benefits in patients with hematoma-type injuries as opposed to those with diffuse injury and contusions.
Zhu [72]	2016	18	2177	TH failed to demonstrate a decrease in mortality and unfavorable clinical outcomes at 3 or 6 months post-TBI. TH might increase the risk of developing pneumonia and cardiovascular complications.
Crompton [73]	2017	41	3109	TH was associated with an 18% reduction in mortality and a 35% improvement in neurologic outcome. A minimum of 33 °C for 72 h, followed by spontaneous, natural rewarming, is optimal. TH is likely a beneficial treatment following TBI in adults but cannot be recommended in children.
Lewis † [74]	2017	37	3110	Heterogeneity was evident in the trial designs and participant inclusion. There is insufficient good-quality evidence that TH will reduce the incidence of death or severe disability or increase the incidence of pneumonia.
Leng ‡ [75]	2017	7	1331	The effects of TH on TBI were heterogeneous. TH seems to provide good outcomes on focal lesions, and in adult patients, Asian patients, and at a relatively higher temperature (33–36 °C).
Zang [76]	2017	21	2302	TH was associated with a significant reduction in mortality. However, the pooled data from five recent studies after 2010 showed that TH increased mortality. The studies before 2010 showed that TH improved neurological outcomes, but the ones after 2010 did not obtain this conclusion.
Watson [77]	2018	22	2346	RCTs with a low-risk bias show significantly more mortality and poor outcomes in the TH group, whereas RCTs with a high-risk bias show the opposite. Low-risk-of-bias studies showed no significant difference in new pneumonia, whereas high-risk-of-bias studies suggested significantly more new pneumonia in the TH group. Avoiding fever and the timing of TH implementation may be important. TH may be more beneficial for evacuated mass lesions.
Olah [78]	2018	14	1786	The analysis of methodologically homogenous studies showed that cooling improves the outcome of severe TBI, and this beneficial effect depends on certain cooling parameters and on their integrated measure, the cooling index. Milder and longer cooling and slower rewarming speeds than 0.25 °C/h are the most important to improve the outcome.
Chen [79]	2019	23	2796	TH did not reduce, and may even increase, the mortality rate of patients with TBI in some high-quality studies. TBI patients with ICP could benefit from TH instead of prophylaxis when initiated within 24 h.
Huang [80]	2020	15	2523	TH can improve long-term neurological recovery (RR = 1.20, 95% CI = 1.01–1.42, *p* = 0.04) for patients with severe TBI, but this does not help decrease mortality. TSA indicated that more studies should be conducted.
Kim § [81]	2020	14	2670	High-quality randomized evidence indicates that TH is associated with higher mortality and no difference in good neurologic outcomes compared with normothermia. TH was associated with a significant increase in arrhythmias. TH would better be avoided outside the settings indicated by the international guidelines.
Wu [82]	2021	6	1207	The use of early prophylactic TH (within 6 h after injury) is not supported as a neurological protection strategy in adult patients with TBI, irrespective of the short-term or long-term. No significant benefits were found regarding hypothermia with different rewarming rates.
Olah [83]	2021	13	1696	Including the POLAR results in the cooling index-based meta-analysis strengthened the conclusion that TH has a significant beneficial effect on the death rate in severe TBI, but only when the cooling index is sufficiently high.

* including 4 induced hypothermia, 4 hypothermia on admission, 1 controlled normothermia, and 7 fever/naturally occurring temperature. † including studies on pediatric TBI. ‡ including 1 study for child TBI. § including 6 with adult TBI, 3 with child TBI, 3 with stoke, 1 with sepsis, and 1 with bacterial meningitis. Abbreviations. CI, confidence interval; GOS, Glasgow Outcome Scale; ICP, intracranial pressure; RCT, randomized controlled trial; RR, risk ratio; TBI, traumatic brain injury; TH, therapeutic hypothermia; TSA, trial sequential analysis.

## Data Availability

Not applicable.

## Supplementary Materials

The following supporting information can be downloaded at: https://www.mdpi.com/article/10.3390/jcm13144221/s1, Table S1: Risk of bias assessment.
