# Supplementary material for: Clinical Insights and Future Directions in Hypothermia for Severe Traumatic Brain Injury: A Narrative Review
Source: J Clin Med. 2024 Jul 19;13(14):4221. doi: 10.3390/jcm13144221 (PMC11278030; doi:10.3390/jcm13144221)
Supplement: Supplementary file 1 [file jcm-13-04221-s001.zip › jcm-2985291-supplementary Table S1.pdf]

	Allocation sequence	Allocation concealment	Blinding	Incomplete outcome data	Selective outcome reporting	Other bias
NABISH:H1	?	?	+	+	?	?
NABISH:H2	+	+	+	+	?	?
BHYPO	+	+	+	+	+	?
HOPES	+	+	+	+	+	?
